# Multivalent and multifunctional polysaccharide-based particles for controlled receptor recognition

**DOI:** 10.1038/s41598-018-32994-y

**Published:** 2018-10-03

**Authors:** Haohao Duan, Mark Donovan, Aude Foucher, Xavier Schultze, Sebastien Lecommandoux

**Affiliations:** 1 L’Oréal recherche avancée, 1 avenue Eugène Schueller, 93600 Aulnay-sous-Bois, France; 2 Laboratoire de Chimie des Polymères Organiques, CNRS, Université de Bordeaux INP/ENSCBP, 16 avenue Pey Berland, 33600 Pessac, France

## Abstract

Polysaccharides represent a versatile class of building blocks that are used in macromolecular design. By choosing the appropriate saccharide block, various physico-chemical and biological properties can be introduced both at the level of the polymer chains and the resulting self-assembled nanostructures. Here, we synthetized amphiphilic diblock copolymers combining a hydrophobic and helical poly(γ-benzyl-L-glutamate) PBLG and two polysaccharides, namely hyaluronic acid (HA) and laminarin (LAM). The copolymers could self-assemble to form particles in water by nanoprecipitation. In addition, hybrid particles containing both HA and LAM in different ratios were obtained by co-nanoprecipitation of the two copolymers. By controlling the self-assembly process, five particle samples with different morphologies and compositions were developed. The interaction between the particles and biologically relevant proteins for HA and LAM, namely CD44 and Dectin-1 respectively, was evaluated by surface plasmon resonance (SPR). We demonstrated that the particle-protein interaction could be modulated by the particle structure and composition. It is therefore suggested that this method based on nanoprecipitation is a practical and versatile way to obtain particles with controllable interactions with proteins, hence with the appropriate biological properties for biomedical applications such as drug delivery.

## Introduction

Polysaccharides represent an important class of polymers for the design of functional biomaterials, especially towards biomedical^1–4^ and cosmetic applications^5–7^. Compared to synthetic polymers, they provide better biocompatibility, biodegradability, and also diverse bioactivities depending on their structures^8–12^. Furthermore, as bio-sourced polymers, polysaccharides are fully renewable ingredients, which make them particularly relevant in the context of green chemistry^13–15^. In addition, their production can be completed in an eco-friendly way with minimal environmental impact, which perfectly matches the criteria of sustainable development for industry.

Among all the available polysaccharides, hyaluronic acid (HA), a non-sulfated glycosaminoglycan (GAG), is composed of alternative units of D-glucuronic acid and N-acetyl glucosamine linked by β-1,3 and β-1,4 glycosidic bonds. HA is one of the main components of the extracellular matrix (ECM), and as such, is highly abundant in the ECM-rich tissues such as synovial fluid, vitreous body of eyes or dermis^16,17^. With a highly hydrophilic structure, HA plays important roles for water retention in skin and eyes^18,19^. In addition, the molecular weight of native HA being large (>1000KDa), the prepared solutions are generally very viscous with a shear-thinning character^20^. These properties make HA the unique space-filling material in tissues to maintain their morphology and homeostasis^21^. This is also the reason why HA is widely used in the dermal fillers for plastic surgery^5^.

In addition to the functions related to its physicochemical properties, HA has numerous biological effects, especially *via* its specific interaction with HA binding proteins (hyaladherins)^22^, such as CD44, RHAMM, Stabilin-2, LYVE-1 and aggrecan. The interaction between HA and CD44 has been widely explored, mainly because CD44 is a glycoprotein expressed at the surface of most cell types including skin keratinocytes^23^ and fibroblasts^24^, and is involved in a number of signal transduction pathways^25^. By interacting with CD44, HA exhibits activities in biological processes such as cell proliferation/migration^26^, wound healing^27^ and tissue regeneration^28^. Compared to other hyaladherins, CD44 attracts researchers’ high interest due to its involvement in cancer^29^. Overexpression of CD44 can be observed in cancer cells including breast, pancreas, gastric, prostate, ovarian and colon^30–35^, making CD44^+^ a biomarker of cancer cells^36^. Targeting overexpressed CD44 in cancer is becoming one of the important strategies in cancer therapy^37^. As the main ligand of CD44, HA is now increasingly used in the nanomaterial design for drug delivery^38^. It has been demonstrated that the interaction of HA with targeted proteins and the relevant effects depends on its molecular weight. Indeed, native HA with high molecular weight in ECM contributes to skin integrity and prevents inflammation and angiogenesis, whereas fragmented HA is generated and involved in a range of immunological processes during the tissue injury and inflammation^39–41^. LAM is another important oligosaccharide that may present relevant biological activity. Indeed, as a β-D-glucan, LAM is a ligand of dectin-1^42^, a pattern-recognition receptor of immune system involved in immune response initiation during fungal infection^43,44^. By interacting with dectin-1, LAM can be used to modulate immune system and bring biological activities including immunostimulatory and antitumorous effects^45,46^.

In this study, particles were designed by self-assembly of polysaccharide-*b*-polypeptide block copolymers. Based on the synthesis strategy previously established in our group^47,48^, two copolymers, based on HA and LAM, were obtained. We further developed a nanoprecipitation process to obtain a range of particles with different morphologies and compositions, including particles containing only HA or LAM and hybrid particles containing both of them in different ratios. The interaction with CD44 was measured by using surface plasmon resonance (SPR). We investigated the interaction of the particles with CD44 in comparison with linear HA of different molecular weights and CD44. We demonstrated that our particles exhibit an enhanced interaction with CD44 compared to linear HA, and that the particle-CD44 interactions could be modulated by changing the ratio of HA in the hybrid particle. Furthermore, a practical method was developed based on SPR to observe the dual functionality of the hybrid particles and their ability to associate simultaneously with CD44 and Dectin-1. Altogether, our experiments demonstrated that our unique copolymer design and self-assembly approach allowed both for an increase of ligand efficacy and dual functionality of particles.

## Result and Discussion

### Polysaccharide-*b*-polypeptide block copolymer synthesis

**Figure 1:**
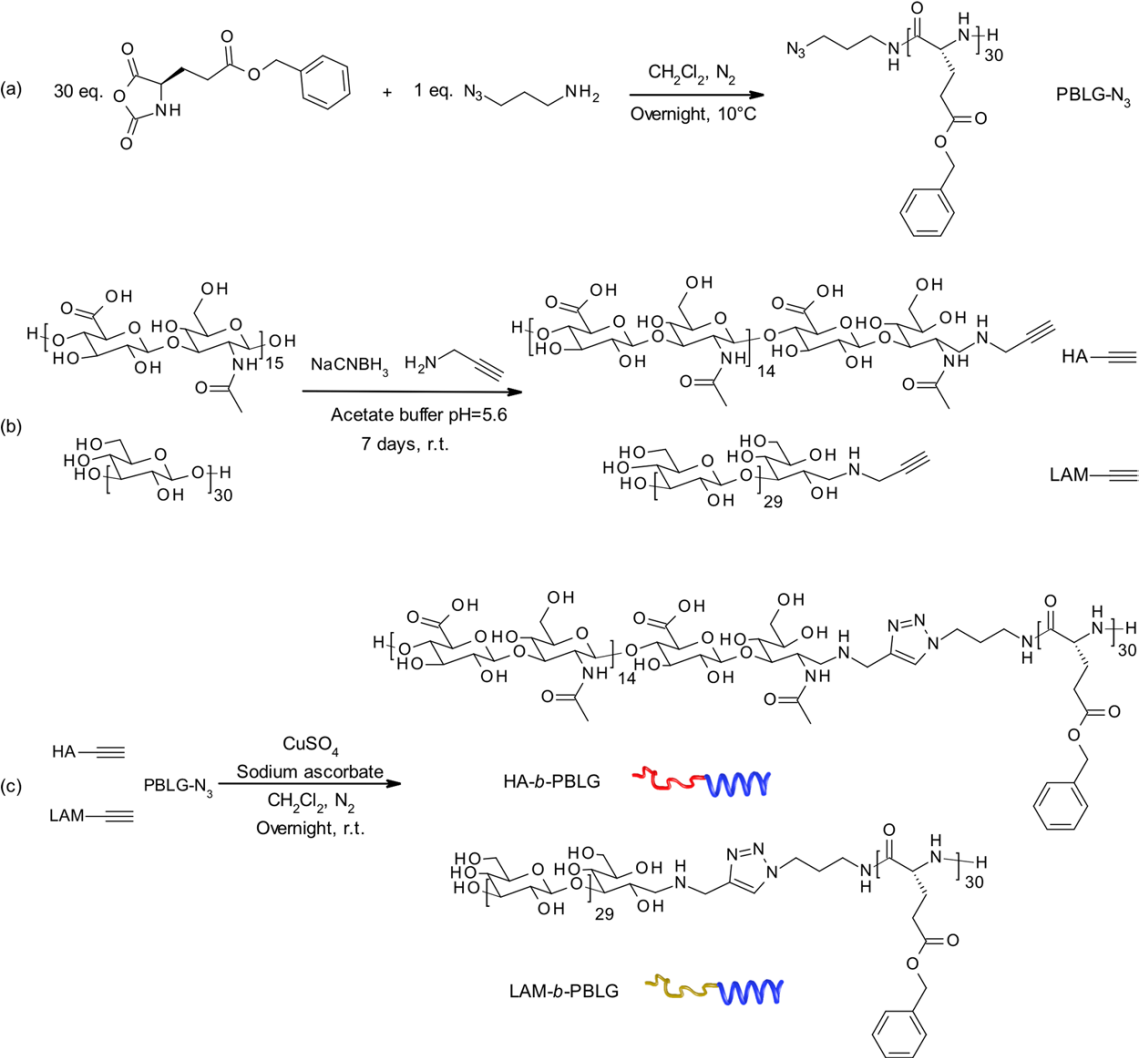
(**a**) Synthesis of poly(γ-benzyl-L-glutamate) by ring-opening polymerization of NCA from azidoaminopropane (**b**) Hyaluronic acid and laminarin functionalization by reductive amination (**c**) Polysaccharide-*b*-polypeptide diblock copolymer (HA-*b*-PBLG, LAM-*b*-PBLG) synthesis by Cu(I)-catalyzed Huisgen cycloaddition.

A three-step synthesis based on copper-catalyzed Huisgen cycloaddition “click chemistry”, previously reported by our group, was developed to obtain the polysaccharide-*b-*polypeptide di-block copolymer^47^. A poly(γ-benzyl-L-glutamate) with azide functional group (PBLG-N_3_) was obtained by the ring-opening polymerization of the corresponding N-carboxyanhydride (NCA) initiated by 3-azido-1-propanamine primary amine (Fig. 1a). This controlled and living polymerization process led to the formation of PBLG-N_3_ with a degree of polymerization DP = 30 (corresponding to 6.6 kDa) and low dispersity (*Đ* = 1.1). Propargyl functionality was introduced to the reducing end of both HA (5 kDa) and LAM (5 kDa) by reductive amination with propargylamine (Fig. 1b). Besides HA^49^ and LAM^50^, other polysaccharides such as xylan, pullulan, dextran or galactan can also be functionalized in the same way^51–55^. The two building blocks were then associated by Cu(I)-catalyzed Huisgen cycloaddition as shown in Fig. 1c to obtain the expected di-block copolymers: HA-*b*-PBLG and LAM-*b*-PBLG.

Chemical modifications of polysaccharides are often introduced on the lateral functional groups on their backbones such as –OH and –COOH. However, the modification ratio can only be controlled statistically and consequently brings additional variability to the resulting product. Furthermore, the biological activities of polysaccharides can be significantly altered by such side chain modifications^56,57^. We can notice that the structure of HA and LAM in our block copolymer is nearly intact compared to its natural structure. Each polysaccharide chain is modified once on the reducing end and the full conversion can be confirmed by ^1^H-NMR. In this way, the chemical structure of the copolymer can be precisely controlled. The resulting nanostructures based on these copolymers can also maintain the properties of HA and LAM to associate with their target biological receptors, respectively CD44 and dectin-1, as shown later in the SPR study.

### Amphiphilic copolymer self-assembly and particle formation

**Figure 2:**
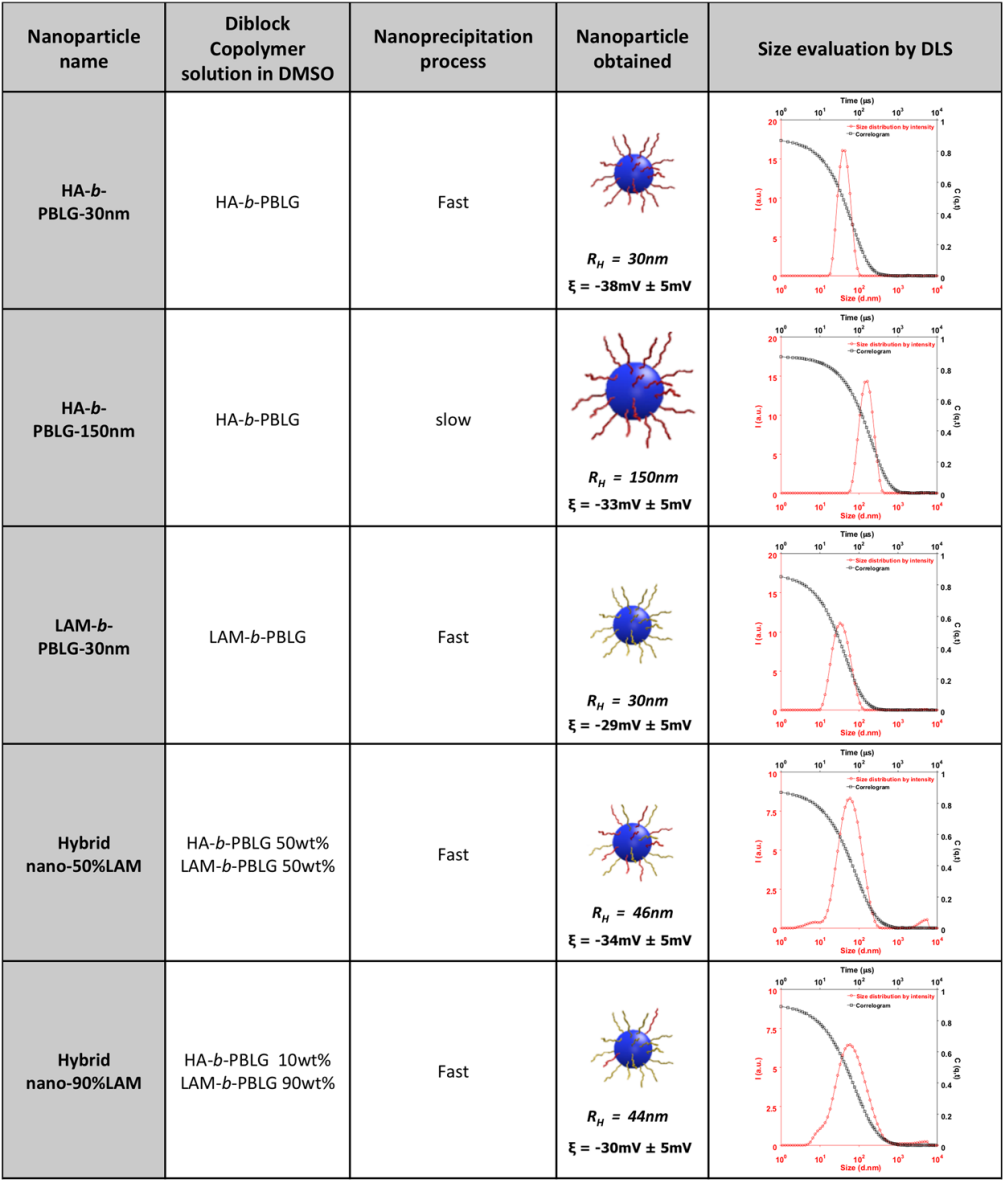
List of diblock copolymers based particles obtained by nanoprecipitation and their characterization (Hydrodynamic radius R_H_ and zeta potential ξ, n = 3).

The two synthesized amphiphilic copolymers HA-*b*-PBLG and LAM-*b*-PBLG were self-assembled in water by using a nanoprecipitation approach, adapted from previous reports^47,58^. First dissolved in DMSO, a good solvent for both blocks, the copolymer solution was then diluted with water. The self-assembly was driven by the insolubility of the hydrophobic PBLG segments in water, that formed the particle core during solvent-displacement. Covalently attached to the PBLG in the copolymer structure, the hydrophilic polysaccharide moieties remained at the particle surface. In this way, we obtained stable self-assembled particles in water. The particle morphology could be modulated by process parameters such as mixing rate and water phase composition^59^. In our study, two process protocols, named fast nanoprecipitation and slow nanoprecipitation, have been optimized, as detailed in the Method section. Three monofunctional particle samples were thus obtained: HA-*b*-PBLG-30nm, HA-*b*-PBLG-150nm, LAM-*b*-PBLG-30nm as listed in Fig. 2.

The two copolymers have similar molecular structures, with the same ratio between the hydrophilic polysaccharide block and the hydrophobic polypeptide block. The resulting particles obtained by fast nanoprecipitation had consistently very similar sizes between 30 nm and 50 nm. In this study, we have identified the experimental conditions to co-nanoprecipitate HA-*b*-PBLG and LAM-*b*-PBLG copolymers together from their mix solution in DMSO during a fast nanoprecipitation process. Indeed, in these conditions, the particles were rapidly formed and instantly “frozen” during the solvent displacement. Such an out-of-equilibrium process favors the homogeneous mixture of the two copolymers in the particles, since the potential phase separation between the copolymers is minimized. The resulting hybrid particles were stable in water, containing both HA and LAM moieties. By changing the initial concentration of each copolymer from the DMSO solution, the composition of the particle can be continuously modulated. In the present study, after extensive dialysis against pure water in order to remove any trace of DMSO, particles with the same size range (around 40 nm), with relatively low polydispersity (PDI < 0.2) and negative zeta-potential (ξ ranging from −38 to −29 mV), but with composition of 90 wt% and 50 wt% of laminarin were obtained, and named Hybrid-nano 90%LAM and Hybrid-nano 50%LAM respectively (Fig. 2). All the relevant characteristics of the developed particles that will be further studied are summarized in Fig. 2.

### Interaction of HA free chains and HA-based particles with CD44 observed by SPR

**Figure 3:**
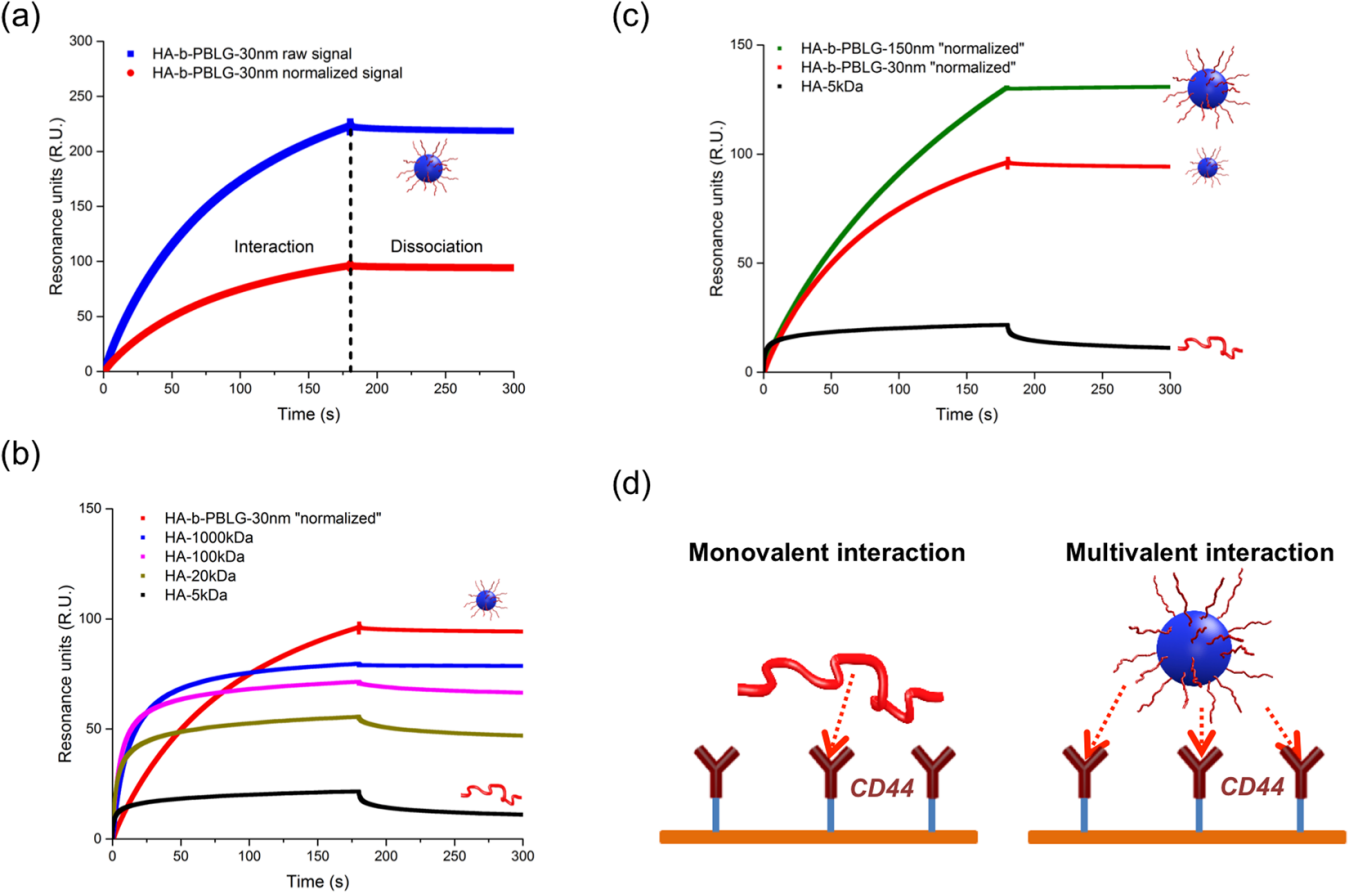
(**a**) Normalization of the particle SPR signal through multiplying by 0.43 to remove the contribution of PBLG block (**b**) Enhanced interaction with CD44 of the HA particle compared to the free HA demonstrated by SPR in normalized condition: HA samples were tested at 10ppm, and HA-*b*-PBLG-30nm was tested at 23ppm in which 10ppm HA was contained (**c**) Influence of the particle size on the interaction with CD44 demonstrated by SPR (**d**) Schematic representation of the multivalent interaction between the HA-particle and CD44.

Previously reported studies exhibited the capability of HA conjugates and particles functionalized with HA to recognize CD44 receptors^60–64^. In our group, polymer vesicles based on HA-*b*-PBLG have been proven efficient to target the overexpressed CD44 in cancer cells and deliver the loaded actives to limit cancer progression^65–67^. In the present study, the interaction between CD44 and HA-based compounds (HA of different molecular weights and the HA-containing particles) was investigated in detail by surface plasmon resonance (SPR). All the analyses were performed in non-saturated conditions, meaning that the CD44 receptors were never fully occupied by the sample. In the block copolymer, the molecular weight of the polysaccharide moiety was 5 kDa and that of polypeptide is 6.6 kDa. As a result, the copolymer and the particles formed by the copolymer, contained only 43 wt% polysaccharide, which is the active moiety to interact with the receptors. In a SPR analysis, the binding signal represents the entire mass of compounds attached to the bioreceptor-functionalized surface^68^. In case of particle-receptor interaction study by SPR, the polypeptide (PBLG) blocks, forming the particle cores, contributed to the SPR signal once the particle was attached to the surface, even they were not involved in the interaction with the receptors. In order to subtract the contribution of PBLG, the SPR signal of the particles was normalized by multiplying by 0.43, the polysaccharide weight ratio in the particle as shown in Fig. 3a. The resulting normalized signal revealed uniquely the quantity of bioactive polysaccharide involved in the interaction. However, the mass percentage of the particle samples (23ppm) in the tests was larger than that of HA samples (10ppm), so that all the samples contained the same quantity of active moiety for the interaction, which was the polysaccharide (Fig. 3b). It is important to notice that all the comparisons in this study were performed in these “double normalized conditions”, meaning that the samples that we used contained the same concentration of HA, and we compare the quantity of normalized SPR signals revealing the quantity of HA associated with the surface by CD44.

As shown in Fig. 3b, the interaction between HA and CD44 increases with the molecular weight of HA, observed by enhanced SPR binding signals. The SPR signal of a high molecular weight HA (1000 kDa) was almost 5-fold stronger than that of a small molecular weight HA (5 kDa). These observations are consistent with previous studies from Dan Peer’s group, also obtained by SPR^69^, and that of Ritchter and coll. through quartz crystal microbalance (QCM-D) analysis^70^. To synthesize the HA-*b*-PBLG copolymer, a low molecular weight HA (5 kDa) was used and consequently the resulting particles were covered with the same units of HA-5kDa. In SPR analysis (Fig. 3b), the particle sample showed a much stronger interaction with CD44 compared to a HA-5kDa sample with the same HA concentration. Even with slower kinetics, the binding signal of the particle sample was even higher than that of the highest molecular weight HA samples (100 kDa and 1000 kDa). With the same HA-*b*-PBLG copolymer, the particle interaction with CD44 could be modulated by its morphology, and therefore by the formulation process. Indeed, as observed in Fig. 3c, the SPR signal of larger HA-particles (150 nm) was significantly higher than that of those of smaller diameters (30 nm). These observations are consistent with recent *in vitro* experiments performed on different lung cancer cell lines expressing different level of CD44^71^. Richter’s lab also recently observed the multivalent interaction between CD44 and high molecular weight HA by a multivalent interaction model^72^. They especially demonstrated by single molecule force spectroscopy that CD44/HA bonds have a high tensile strength despite their low affinity, and that multiple bonds along an HA chain rupture independently under load. Our experiments suggest that this interaction enhancement with the HA particle performed with the same principle. As schematically illustrated in Fig. 3d, a free HA-5kDa moiety in the solution can probably associate with only one or a very low number of CD44, whereas the HA particles were able to bind simultaneously a larger number of CD44. These multivalent interactions strengthen the interfacial force, and hence the binding signal in SPR analysis. This was also the reason why we were unable to measure the association and the dissociation constants. Indeed, to obtain these constants by curve fitting algorithms, it is essential to know the precise interaction model (1:1, 1:2 etc.) between the receptors and the analytes in the sample, which is very challenging with any particle systems.

### Interaction modulation by controlling the particle composition

**Figure 4:**
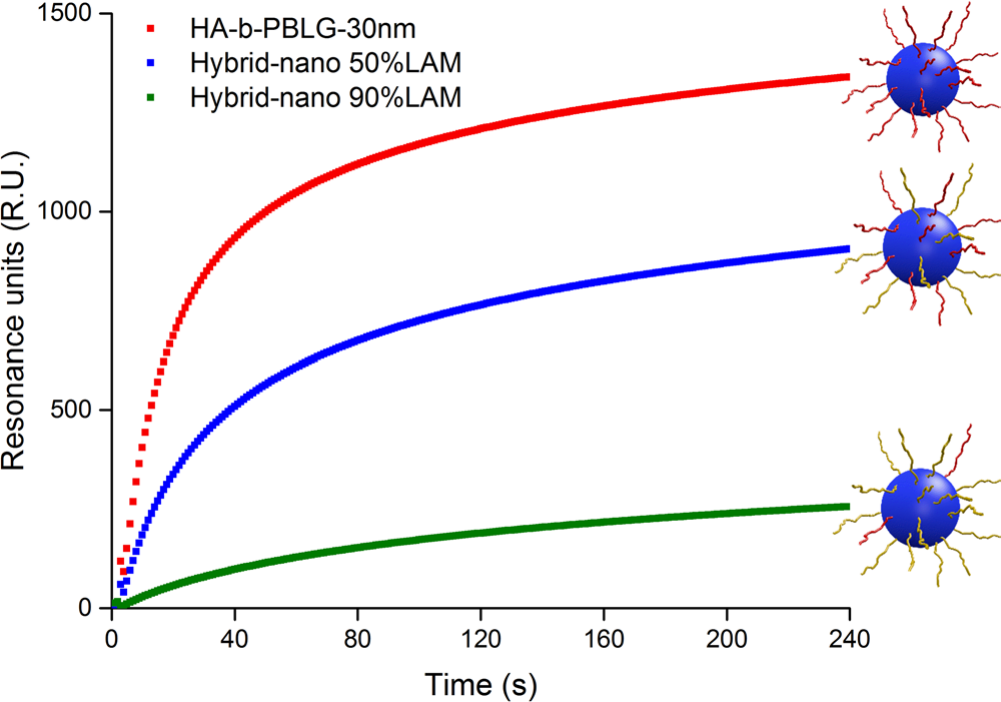
Modulation of interaction strength between the hybrid particles and CD44 observed by SPR.

The interaction of the three HA-containing particles with CD44 was further compared with different HA content toward LAM (meaning HA-*b*-PBLG-30nm, Hybrid-nano 50%LAM and Hybrid-nano 90%LAM). All of them presented significant interaction signals with CD44, but the binding level was reduced with the decreasing ratio of HA in the particle as shown in Fig. 4. The three different particle samples were obtained by the same fast nanoprecipitation with similar sizes (Fig. 4), so that they were able to get in contact with a similar quantity of CD44 on the SPR surface. However, LAM was unable to associate with CD44. By introducing LAM on the particle surface, the density of HA on each particle was reduced, and so was the multivalency degree of the interaction with CD44 as well. As a consequence, the interaction strength with CD44 can be weakened by this dilution and modulated by the ‘dilution’ factor, which explains the SPR signal reduction in Fig. 4.

### The dual functionality of the hybrid particle confirmed by SPR

**Figure 5:**
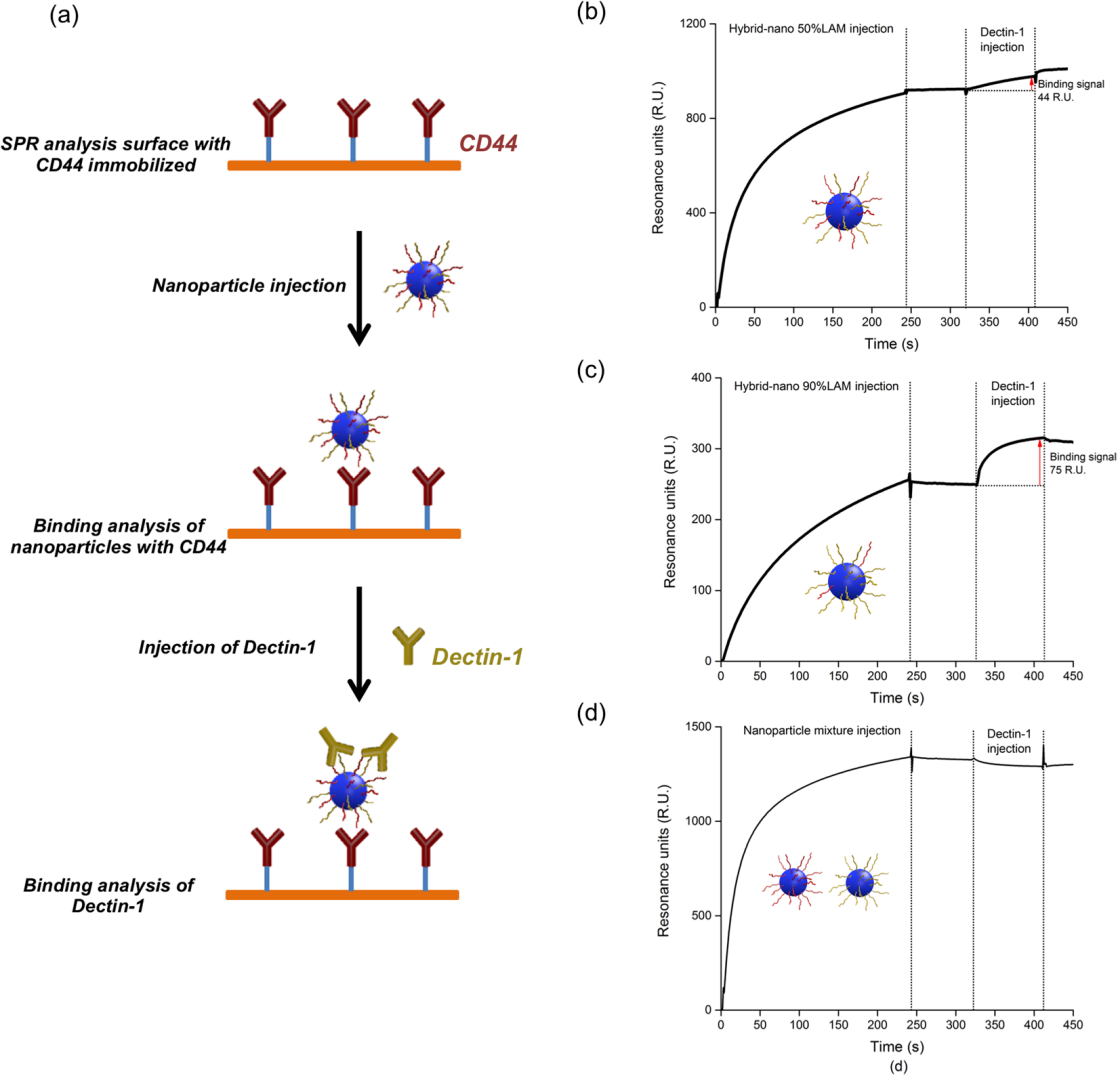
(**a**) Schematic representation of the bi-functional assay as performed by SPR. (**b**) Dual functionality of Hybrid-nano 50%LAM particle demonstrated by the bifunctionality assay. (**c**) Dual functionality of Hybrid-nano 90%LAM particle demonstrated by the bifunctionality assay. (**d**) No dual functionality was observed with a monofunctional particle mixture in the assay: absence of binding signal during dectin-1 injection.

The hybrid particles were obtained by co-nanoprecipitating the both HA-*b*-PBLG and LAM-*b*-PBLG copolymers from a common solution. By formulating polysaccharide moieties in a nanomaterial, it is possible to modulate the bioactivities of the resulting structure such as biological process regulation and inflammatory responses^73^. However, it was important to confirm that the two copolymers self-assemble together in our process and the resulting particle sample was not a mixture of the monofunctional particles. For this purpose, a two-step assay based on SPR was designed to check the coexistence of HA and LAM on the hybrid particle surface, hence its dual functionality. As illustrated in Fig. 5a, the CD44 surface in this assay can interact with the HA moieties and capture the hybrid particle in the first step. A short natural dissociation step was applied to confirm the stable interaction between the sample and the CD44 surface. Then, a second injection was directly performed with dectin-1, a specific biological receptor of LAM^44^. Dectin-1 can associate with LAM units on the hybrid particle but not with HA, and form the sandwich-like structure shown in Fig. 5a. The adsorption of dectin-1 at the surface of the particles can generate a positive binding signal during the injection. As shown in Fig. 5b,c, both hybrid particle samples gave positive binding signals as expected during the two injection steps. Meanwhile as a negative control, the same assay was applied to a mixture of monofunctional particles based on HA and LAM (Fig. 5d). The CD44 surface captured the HA particles and gave a binding signal during the first injection. However, no binding signal was observed during the second injection of Dectin-1, since no LAM was present on the surface of the HA particles. These observations really proved that (i) only particles with HA can bind to CD44 and that there is no unspecific adsorption of LAM based particles, and (ii) the LAM polysaccharide chains present at the surface of hybrid particles are still available for interaction with Dectin-1. By increasing the ratio of LAM on the particle surface, the interaction signal with CD44 decreased whereas that with dectin-1 increased. Through this assay, the dual functionality of the hybrid particles to interact simultaneously with CD44 and dectin-1 is confirmed and thus the concept of interaction modulation by changing the particle composition.

In summary, we have reported a versatile strategy to design particles with tunable interaction with targeting proteins by the combination of different amphiphilic copolymers in a nanoprecipitation process. Using two different polysaccharide-*b*-polypeptide copolymers, namely HA-*b*-PBLG and LAM-*b*-PBLG, a broad range of particles was obtained by changing the formulation process parameters. The control of the nanoprecipitation and co-nanoprecipitation processes allows the design of particles with controlled sizes and compositions in an accurate and reproducible manner. As demonstrated using SPR as a fast and efficient screening method, the interaction of the HA-particle with CD44 was far stronger than that of linear HA on its surface, which can be explained by a multivalent interaction between HA segments on the particle surface and CD44. The “multivalent-degree” can be controlled by changing the particle morphology and composition, hence leading to the modulation of the interaction strength. The combination of the two copolymers results in the formation of hybrid particles with the functionalities from both HA and LAM, as confirmed by an original method based on SPR analysis. All these observations and experimental results strongly suggest that such a simple co-nanoprecipitation process can be a versatile and practical way to design multifunctional particles with tunable biological activities.

## Materials

Hyaluronic acid (HA) sodium salt of different molecular weights (research grade, HA-5kDa, HA-20kDa, HA-100kDa, HA-1000kDa) was purchased from LifeCore Biomedical (Cheska, MN, USA). Laminarin from Laminaria Digitata and all the chemicals used in the copolymers synthesis were purchased from Sigma Aldrich (St. Louis, MO, USA). Recombinant human CD44 and dectin-1 were purchased from R&D system (Minneapolis, MN, USA). CM5 chip and all the solvents and reagents for SPR analysis including the running buffer, N-hydroxysuccinimide (NHS), ethyl-3(3-dimethylamino)propylcarbodiimide (EDC), 1 M ethanolamine solution pH 8.5, the regeneration solution were purchased from GE Healthcare (Uppsala, Sweden) and used as suggested. Ultrafiltration discs were purchased from EMD Millipore (Billerica, MA, USA).

## Measurements

^1^H-NMR spectra were obtained with a Bruker Avance 400 MHz spectrometer (Rheinstetten, Germany). Dynamic light scattering was measured Malvern Zetasizer NANO ZS (Worcestershire, UK). Surface plasmon resonance analysis was performed with Biacore T200 (Uppsala, Sweden). IR spectra were recorded on Perkin Elmer Spectrum One FT-IR (Shelton, CA, USA).

## Methods

### Synthesis of HA-*b*-PBLG and LAM-*b*-PBLG

HA-*b*-PBLG was prepared as reported elsewhere^47^, and LAM-*b*-PBLG was prepared using the same synthetic approach. The polysaccharide blocks have a 5 kDa molecular weight and the same PBLG of 6.6 kDa was used for the click reactions. The copolymers were analyzed by ^1^H-NMR and IR spectroscopy.

### Particle preparation by nanoprecipitation-induced self-assembly

As mentioned in Fig. 2, the experimental conditions of two processes, fast and slow nanoprecipitation, have been optimized as reported below:

Fast nanoprecipitation: 9 ml PBS buffer (10 mM, pH = 7.4, 154 mM ionic strength) was heated to 50 °C and stirred at 500 rpm by a magnetic rotor. 1 ml copolymer solution in DMSO (1 wt%), previously heated to 50 °C, was added dropwise to the PBS buffer. The resulting solution was further stirred at 50 °C for 30 min then cooled down to room temperature. DMSO was removed by ultrafiltration with the PBS buffer against a MWCO = 100 kDa filter. The particle size was measured by DLS with a scattering angle at 173°.

Slow nanoprecipitation: 1 ml copolymer solution in DMSO (1 wt%) was maintained at 60 °C and stirred at 500 rpm. 9 ml PBS buffer (10 mM, pH = 7.4, 154 mM ionic strength) was added dropwise for 400 seconds by a syringe pump. The resulting solution was further stirred at 60 °C for 30 min then cooled down to room temperature. DMSO was removed by ultrafiltration with the PBS buffer against a MWCO = 100 kDa filter. The particle size was measured by DLS with a scattering angle at 173°.

HA-*b*-PBLG-30nm was obtained by using a 1 wt% solution of HA-*b*-PBLG in DMSO with fast nanoprecipitation.

HA-*b*-PBLG-150nm was obtained by using a 1 wt% solution of HA-*b*-PBLG in DMSO with controlled nanoprecipitation.

LAM-*b*-PBLG-30nm was obtained by using a 1 wt% DMSO solution of LAM-*b*-PBLG in DMSO with fast nanoprecipitation.

Hybrid-nano 90%LAM was obtained by using a DMSO solution containing 0.9 wt% LAM-*b*-PBLG and 0.1 wt% HA-*b*-PBLG with fast nanoprecipitation.

Hybrid-nano 50%LAM was obtained by using a DMSO solution containing 0.5 wt% LAM-*b*-PBLG and 0.5 wt% HA-*b*-PBLG with fast nanoprecipitation.

### SPR analysis: surface functionalization and interaction analysis

CM5, a carboxymethylated dextran sensor chip from GE Healthcare, was used for SPR analysis. Recombinant human CD44 was immobilized on the CM5 chip by using a standard amine coupling protocol of Biacore T200. Briefly, the sensor chip flow cell was activated by an EDC/NHS mixture for 420 seconds. CD44 was dissolved in 10 mM acetate buffer pH 4 at 10 µg/ml then injected to the activated surface for 300 seconds. About 4000RU of CD44 was immobilized on the chip during the injection. The remaining activated positions on the chip were then deactivated by 1 M ethanolamine pH 8.5. A blank flow cell was prepared as a reference by the same protocol without CD44 injection.

HBS-EP+ buffer, proposed by GE Healthcare, was chosen as the running buffer to perform all SPR analysis in this study. All the sample injection was performed at a rate of 30 µl/min. For the study in Fig. 4, the solutions of HA of different molecular weights (5 kDa, 20 kDa, 100 kDa, 1000 kDa) was prepared at 10 ppm in the running buffer, whereas those of HA-*b*-PBLG-30nm and HA-*b*-PBLG-150nm were prepared at 23ppm so that all the samples contain the same quantity of HA for comparison. For the same reason, HA-*b*-PBLG-30nm was prepared at 100ppm, Hybrid nano-50%LAM was prepared at 200ppm and Hybrid nano-90%LAM was prepared at 1000ppm for the study in Fig. 4. The particle samples were injected at 100ppm and dectin-1 solution was added at 10 µg/ml in the study shown in Fig. 5. The responses on the blank flow cell were systematically subtracted from the signal obtained on the CD44 coated flow cell to remove the contribution of unspecific interaction independent from CD44.

The surface was regenerated by adding 50 mM NaOH for 30 seconds after each analysis. The full removal of the analyte attached to CD44 was confirmed by the baseline level after the regeneration.
